# Strengthened Anti-Inflammatory Potential of a Purified Antimicrobial Peptide Extract in Leukocyte–Biomaterial Interactions

**DOI:** 10.3390/ma19194043

**Published:** 2026-09-22

**Authors:** Beata Drzewiecka, Michał Świeca, Dominika Nguyen Ngoc, Agnieszka Furmańczyk-Gnyp, Joanna Wessely-Szponder

**Affiliations:** 1 Department of Pathophysiology, Faculty of Veterinary Medicine, University of Life Sciences, Akademicka 12, 20-033 Lublin, Poland; beata.drzewiecka@up.edu.pl (B.D.); dominika.nguyen@up.edu.pl (D.N.N.); agnieszka.furmanczyk@up.edu.pl (A.F.-G.); 2 Department of Biochemistry and Food Chemistry, University of Life Sciences, Skromna Str. 8, 20-704 Lublin, Poland; michal.swieca@up.edu.pl

**Keywords:** antimicrobial peptides, biomaterial, immunology, hydroxyapatite, blood derived products, inflammation, macrophages, neutrophils

## Abstract

Excessive inflammation following implant insertion may impair early healing and predispose to postoperative complications. Since neutrophils constitute the predominant leukocyte population during the initial inflammatory phase, and monocytes subsequently orchestrate tissue resolution and repair, understanding their direct response to biomaterials and bioactive compounds is essential. In the present study, we evaluated the effect of purified bioactive extract combined with biomaterial on neutrophil and monocyte activity in vitro, comparing their modulatory potential with that of non-purified extract. Porcine neutrophils and monocytes were cultured in contact with hydroxyapatite (HA) mixed with purified fractions obtained through sequential chromatographic isolation. Cellular responses were assessed by measuring enzyme release and reactive oxygen/nitrogen species (ROS/RNS) generation. Neutrophils exposed to HA alone displayed a pronounced oxidative response, confirming strong activation. Purified extracts, however, significantly reduced ROS/RNS production (NO: 2.71 ± 0.07 µM vs. HA-only group 5.72 ± 0.10 µM, *p* < 0.05), whereas non-purified extracts elicited only a partial reduction. Enzyme release followed the same trend, with purified fractions demonstrating a markedly higher inhibitory effect on neutrophil degranulation. Similarly, monocytes exposed to HA exhibited prolonged oxidative stress at 24 h and 48 h, which was markedly downregulated by the purified extract (HA + EX), demonstrating significant reductions in both nitric oxide (NO) and superoxide generation. These findings indicate that purification enhances the bioactivity and specificity of the tested extracts, improving their capacity to modulate excessive neutrophil and monocyte activation. To address this challenge, this study introduces a novel, advanced purification method designed to isolate and refine AMPNE-type particles from the crude extract. Furthermore, we investigated whether the purified particles retain their structural and functional integrity post-purification. By comparing the biological efficacy of the extract before and after purification, we evaluated whether the cleaning process maintains, enhances, or modifies their original bioactive properties, providing crucial insights into the viability of the proposed technology. Our study highlights the potential of purified bioactive molecules to more effectively regulate early inflammatory responses to biomaterials. These results provide a foundation for further research into targeted immunomodulatory strategies aimed at improving implant integration and reducing inflammatory complications.

## 1. Introduction

Neutrophils, comprising about 50–70% of circulating leukocytes, are the most abundant immune cells in humans and originate in the bone marrow from myeloid precursors through a process called granulopoiesis. This differentiation is primarily regulated by cytokines, especially granulocyte colony-stimulating factor (G-CSF) [1]. While traditionally considered short-lived, neutrophils can have extended life spans following tissue damage due to the temporary upregulation of antiapoptotic genes. Upon injury, neutrophils are the first immune responders, activated through the release of various signals, including endogenous damage-associated molecular patterns (DAMPs), anaphylatoxins (e.g., C3a and C5a), and chemotactic cytokines such as IL-8 [2]. They possess high microbicidal capabilities, equipped with numerous receptors to identify pathogens, and utilize a combination of cytotoxic mechanisms, such as the production of reactive oxygen species (ROS), neutrophil extracellular traps (NETs), inflammatory mediators, and proteolytic enzymes. Historically, neutrophil activity was viewed as potentially damaging to surrounding tissues; however, recent advancements, including intravital microscopy, genetic fate mapping, transgenic models, and single-cell sequencing, have reshaped this understanding, revealing neutrophils’ critical roles in resolving inflammation and facilitating tissue repair through anti-inflammatory, pro-angiogenic, and regenerative functions [3]. There is a close functional similarity between porcine and human neutrophils; consequently, the pig serves as a suitable animal model for research [4].

Monocytes have transcended their historical classification as mere circulating precursors to tissue macrophages, emerging as central, context-dependent arbiters of the inflammatory cascade whose functions are profoundly shaped by a convergence of biochemical, biophysical, and developmental cues [5]. The functional plasticity of monocytes is exquisitely sensitive to the physical properties of their microenvironment, with extracellular matrix stiffness and hemodynamic shear stress acting as potent rheostats that tune their adhesion, migration, and polarization, thereby directly linking tissue mechanobiology to immune function [6]. The influence of aging further complicates this picture, with the process of “inflammaging” driving a myeloid-biased hematopoiesis, expanding non-classical (CD14^+^CD16^++^) subsets, and creating a state of premature immunosenescence, as observed in chronic conditions like rheumatoid arthritis, where monocyte dysregulation fuels a self-perpetuating cycle of inflammation [7]. Intriguingly, this dynamic interplay extends to novel signaling pathways; Wnt signaling, for instance, has been shown to directly stimulate cytokine and chemokine secretion from human primary monocytes, enhancing their migratory capacity via CCL2 and illustrating how core developmental pathways can be co-opted to drive inflammatory processes in the circulation [8]. This age-related drift not only correlates with poor cardiovascular outcomes in the elderly but is also markedly accelerated in autoimmune conditions such as rheumatoid arthritis, wherein premature immunosenescence exacerbates synovial inflammation and fuels the erosive joint disease [9].

Collectively, these insights position monocytes not as simple effector cells, but as sophisticated, multi-sensory integrators that decode inflammatory, mechanical, and aging-related signals, making them both a sentinel readout of tissue status and a compelling, though complex, therapeutic target for modulating inflammation across a spectrum of diseases.

Arguably the most transformative conceptual advance in contemporary monocyte research resides in the elucidation of “trained immunity,” a de facto functional state of innate immune memory that overturns the classical dogma of immunological amnesia in myeloid cells. Mechanistically, training is initiated by primary exposure to prototypical microbial ligands such as β-glucan or the live-attenuated BCG vaccine, which instigates a protracted reprogramming of intracellular metabolism, typified by a shift toward aerobic glycolysis, accumulation of the tricarboxylic acid cycle intermediate succinate, and enhanced cholesterol synthesis via the mevalonate pathway. These metabolic perturbations are inextricably coupled with enduring changes in the epigenetic landscape, including sustained deposition of permissive histone marks such as trimethylation of histone H3 at lysine 4 (H3K4me3) and acetylation at lysine 27 (H3K27ac) at the promoters of pro-inflammatory cytokine genes [6]. Crucially, this reprogramming is not ephemeral or restricted to post-mitotic circulating cells; rather, it is propagated retrograde to the hematopoietic stem and progenitor cell (HSPC) compartment within the bone marrow niche, establishing a “central” trained state that persists for months and systemically amplifies myeloid output and inflammatory responsiveness upon secondary challenge [7,10]. Furthermore, an emerging frontier in the field concerns the mechanobiological sensitivity of monocytes; these cells are exquisitely capable of perceiving and transducing physical forces from their microenvironment, including variations in extracellular matrix (ECM) stiffness and hemodynamic shear stress. This mechanotransduction operates through integrin-dependent focal adhesion signaling and cytoskeletal remodeling, ultimately converging on the activation of the transcription regulators YAP/TAZ, which in turn modulate monocyte adhesion dynamics, migratory persistence, and the delicate balance between M1-like pro-inflammatory and M2-like tissue-reparative polarization states. Such mechano-responsiveness is of particular relevance in fibrotic disorders and in the distinct hemodynamic zones of arterial bifurcations, where disturbed flow patterns actively promote a pro-atherogenic monocyte phenotype [11].

Taken together, the current state of knowledge positions monocytes not as simple effector cells, but as sophisticated, integrated decision-making hubs that metabolically, epigenetically, and mechanically encode inflammatory experience. Their strategic position at the nexus of innate and adaptive immunity, thrombosis, and tissue remodeling renders them both promising therapeutic targets and formidable challenges, necessitating the development of context-aware, precision immunomodulatory interventions that selectively abrogate pathogenic inflammatory functions while preserving requisite host-defense and tissue-reparative capacities [12].

For these reasons, we decided to evaluate the activity of neutrophils and monocytes toward an implanted biomaterial combined with a purified peptide extract that we obtained in our previous research [13]. To this end, we evaluated the generation of reactive oxygen and nitrogen species (ROS/RNS) and the secretory response of neutrophils through enzyme release.

Hydroxyapatite (HA) is a calcium phosphate ceramic characterized by high biocompatibility, osteoinductivity, and osseointegration capability. It has been developed as an implantable material with potential for bone regeneration in different forms such as bone substitutes, scaffolds, and implant coatings, and is widely used in orthopedics and dentistry [14]. However, HA tends to fragment and release small particles, which can activate neutrophils and monocytes during the repair process, potentially resulting in implantation failure. The mechanical parameters and stability of HA are typically enhanced through the creation of composite biomaterials, while its biological properties can be substantially altered by various substitutions and modifications [15].

Host defense peptides (HDPs), commonly referred to as antimicrobial peptides (AMPs), are cationic peptides that demonstrate diverse antimicrobial and immunomodulatory functions; their capacity to facilitate communication among immune cells also enhances healing processes. The activities of AMPs encompass the modulation of specific signaling pathways during inflammation, T-cell responses, the differentiation and polarization of macrophages and dendritic cells, wound repair, and the apoptosis of certain immune cells. At present, certain antimicrobial peptides (AMPs) are undergoing assessment in preclinical studies; for instance, magainin, derived from frog skin, has progressed to phase III of a clinical trial [16,17,18].

The natural peptide mixture known as antimicrobial porcine extract (AMPNE) is derived from fresh porcine blood and comprises various antimicrobial peptides, including protegrins, PR-39, and prophenins. This product, apart from its confirmed antimicrobial activity, plays an important immunomodulatory role [19,20,21]. Based on previous research, AMPNE appeared to be useful in the regulation of the inflammatory response, especially in the presence of or after previous stimulation with biomaterials [13]. Consequently, after achieving promising results previously, we resolved to broaden the research scope to purify that extract. The overactivation of immune cells leads to increased secretion of pro-inflammatory mediators, which may disrupt the repair process. Consequently, novel tissue regeneration strategies that increasingly emphasize the immunomodulatory characteristics of various biomaterials and blood-derived products are garnering heightened interest. According to some authors, different AMPs in the regulation of inflammation should be considered rather as regulators of immune homeostasis than simply as pro-inflammatory or anti-inflammatory factors [20]. Consequently, AMPNE was utilized to stimulate neutrophil and monocyte cultures during incubation with hydroxyapatite combined with a purified extract to evaluate their impact on homologous (porcine) leukocytes.

Consequently, the primary objective of this research was twofold: first, to establish and optimize an innovative, high-efficiency purification protocol for the extraction of AMPNE-type particles; and second, to systematically assess the impact of this downstream processing on their functional properties. Specifically, we sought to determine whether the isolated particles preserve their biological activity or if the purification process alters their efficacy either by eliminating interfering matrix compounds to enhance the observed effect, or conversely, by diminishing synergistic mechanisms present in the crude extract. Demonstrating that the purified fraction retains or strengthens the desired profile is critical for validating the novel extraction method and confirming the true active nature of AMPNE particles.

We hypothesize that the physicochemical characteristics of hydroxyapatite granules, including surface topography, charge, and degradation rate, substantially affect the intensity and nature of the immune response generated. We propose that the immune response to biomaterials progresses over time, characterized by specific phases of inflammation, tissue remodelling, and the possible induction of immune tolerance. The main aim of this study is to thoroughly investigate the immune responses elicited by biomaterial in conjunction with purified extract using an in vitro porcine model.

Given the increasing prevalence of drug resistance, the formulation of a biologically derived substance comprising a blend of peptides with established antimicrobial and immunomodulatory properties will represent a significant advancement in biomaterial sciences and may serve as a therapeutic alternative pending clinical trials.

Building upon our previous findings regarding the leukocytic responses to crude porcine neutrophil peptide extracts [13], the present study specifically focuses on the isolated effects of the purified fraction AMPNE. While the unpurified extract exhibited broad immunomodulatory properties in our prior work, purification was performed to eliminate non-specific protein contaminants and enrich the active peptide component. Therefore, rather than repeating the baseline evaluation of the crude extract, this investigation aimed to directly characterize the targeted anti-inflammatory and modulating capacity of purified AMPNE in co-culture with hydroxyapatite particles.

## 2. Materials and Methods

### 2.1. Biomaterials Used for the Study

Commercially available hydroxyapatite granules (Sigma-Aldrich; St. Louis, MO, USA; 0.5–1.0 mm grain size) were used for the stimulation of porcine neutrophils and monocytes in vitro. Before the experiment, the biomaterial was suspended in sterile phosphate-buffered saline (PBS) on 24-well plates at 37 °C with 5% CO_2_ for 2 h. Commercially available hydroxyapatite is referred to as HA throughout the manuscript. The modified biomaterial was prepared under similar conditions and contained purified peptide extract as a modulating additive. The neutrophil extract content is 0.2% of the biomaterial mass. The modified hydroxyapatite is referred to as HA + EX throughout the manuscript.

### 2.2. Preparation of AMPNE

Neutrophils were isolated from the porcine blood collected via slaughter according to the previously described method [19]. Antimicrobial peptide neutrophil extract (AMPNE) was isolated according to the procedure previously described in our previous work [13].

### 2.3. Purification of AMPNE

The lyophilisates were dissolved in PBS and collected in 15 mL Falcon tubes, 5 mL in each. Then, 0.5 g of NaCl was added and centrifuged (13,000× *g*, 3 min, 4 °C). The supernatant was used for further analysis.

Gel filtration chromatography was used to separate the components according to their size. The extract was passed through a Sephadex G-25 column (Fine, Sigma-Aldrich) using 5% acetic acid buffer at a flow rate of 0.5 mL/min. The absorbance of the eluate (every 0.5 mL) was monitored at 280 nm, and fractions were pooled after 1.5 mL. The concentration of protegrins and other cathelicidins in each fraction was assessed using the extinction coefficient.

### 2.4. Incubation of Porcine Neutrophils with HA With or Without Stimulation with Extract from Purified Peptide

Isolated neutrophils were seeded into culture plates, followed by the addition of HA granules and the subsequent introduction of the purified peptide extract solution. The cells, HA particles, and peptides were co-incubated in the culture medium for 24 h or 48 h. Following incubation, cell-free supernatants were collected for biochemical analyses. Porcine neutrophils were incubated with HA with or without stimulation by purified peptide extract according to the protocol detailed in our previous study [13]. The representative cultures from all groups were additionally stimulated with 40 µg/mL of AMPNE and marked as HA + EX. Neutrophils without biomaterials were the control group, marked as N.

### 2.5. Isolation and Incubation of Porcine Monocytes with HA With or Without Stimulation with Extract from Purified Peptide

Porcine peripheral blood mononuclear cells (pPBMCs) were isolated from porcine blood via Gradisol L density gradient centrifugation as described previously for rabbit macrophages [22]. Porcine monocytes were incubated with HA (with or without stimulation with purified peptide extract) following the protocol detailed in our previous study [13]. Cells were seeded into 24-well plates containing 25 mg of HA or HA modified with purified peptide extract (HA + EX) or incubated without biomaterial as a control (K). Incubation was carried out at 37 °C with 5% CO_2_ for extended time intervals of 24 h and 48 h to reflect sustained mononuclear cell responses.

### 2.6. Evaluation of the Response of Neutrophils and Monocytes to HA, HA + EX

Freshly isolated porcine neutrophils (1 × 10^6^ cells/well) were resuspended in 500 μL of PBS and seeded onto 24-well plates containing 40 mg of the tested biomaterials per well. Incubation was carried out under humidified conditions (37 °C, 5% CO_2_) for 4 h and 24 h. Unstimulated neutrophils cultured without biomaterial served as the baseline control (group N). Where indicated, cell populations were supplemented with 40 µg/mL of homologous extract, with the combination of hydroxyapatite and peptide extract designated as HA + EX.

Similarly, porcine monocytes were resuspended at an identical density (1 × 10^6^ cells in 500 μL of complete culture medium per well) and exposed to 40 mg of biomaterial samples in 24-well plates under standard culture conditions (37 °C, 5% CO_2_) for 24 h and 48 h. Monocytes incubated in the absence of biomaterials constituted the negative control (group K). Parallel treatment groups were additionally challenged with 40 µg/mL of homologous extract, with HA constructs containing AMPNE labeled as HA + EX.

### 2.7. Assessment of Leukocyte Activity In Vitro

Porcine neutrophil activation in response to the tested samples was determined at two time points (4 h and 24 h). To assess azurophilic granule exocytosis, elastase and myeloperoxidase (MPO) enzymatic activities were measured via azocasein degradation (Sigma-Aldrich, St. Louis, MO, USA) using a BioTek EL800 spectrophotometric reader (BioTek, Janki, Poland). Mobilization of secretory vesicles was determined by evaluating alkaline phosphatase (ALP) activity, where cells were reacted for 10 min at 25 °C with 4-nitrophenyl phosphate disodium salt hexahydrate (Sigma-Aldrich, St. Louis, MO, USA). Accumulation of nitrites in the extracellular medium served as an indicator of NO release by porcine leukocytes and was measured via the standard Griess reaction against a known nitrite concentration curve. Superoxide radical formation was assessed by incubating leukocytes with 0.1% nitroblue tetrazolium (NBT) (Sigma-Aldrich, St. Louis, MO, USA) for 15 min at room temperature. Following incubation, absorbance was read at 545 nm, and superoxide content was calculated employing the molar extinction coefficient of NBT (21.1 nMol) [22].

### 2.8. Statistical Analysis

All measurements were performed on three biological replicates, and data are presented as mean ± SD. Comparative evaluations against the control group were executed via one-tailed paired Student’s *t*-test in Statistica version 13.1 (Dell Inc., Tulsa, OK, USA). Statistical significance was defined as *p* < 0.05, whereas *p* < 0.01 was considered highly significant.

### 2.9. Ethics Statement

The present study did not require formal approval from an institutional animal ethics committee, as all biological materials were derived from porcine blood collected during routine, commercial slaughter of animals processed for human consumption at a licensed slaughterhouse. No procedures were performed on live animals, nor were any animals euthanized specifically for the research objectives of this study. Accordingly, the activities conducted do not fall within the scope of animal experimentation as defined by Directive 2010/63/EU of the European Parliament and of the Council on the protection of animals used for scientific purposes, nor under the Polish Act of 15 January 2015 on the Protection of Animals Used for Scientific or Educational Purposes (as amended).

## 3. Results

To evaluate the biological response, leukocytes were divided into specific treatment groups. Neutrophil cultures comprised unstimulated cells (N), cells treated with purified neutrophil peptide extract alone (EX), cells exposed to hydroxyapatite (HA), and cells co-stimulated with hydroxyapatite and the peptide extract (HA + EX). Similarly, monocyte experimental setups were designated as untreated controls (K), hydroxyapatite-treated cells (HA), and monocytes exposed to both hydroxyapatite and the porcine AMPNE (HA + EX). All assays were executed in triplicate, and statistical evaluations were performed using a one-tailed paired Student’s *t*-test, with data expressed as mean ± SD (* *p* < 0.05, ** *p* < 0.01).

Primary granule exocytosis, evaluated via elastase and MPO release (Figure 1 and Figure 2), was significantly upregulated (*p* < 0.05) following exposure to HA at both 4 h and 24 h time points relative to the unstimulated baseline. Assessment of secretory granule mobilization via ALP levels (Figure 3) similarly demonstrated a marked increase upon HA stimulation compared to untreated controls, whereas the effect of HA remained substantially weaker. The inclusion of AMPNE as a co-stimulant consistently attenuated the neutrophil response. Specifically, AMPNE co-treatment significantly suppressed elastase, MPO, and ALP secretion at both time points in HA + EX cultures, with a parallel, albeit less pronounced, inhibitory effect observed in the HA groups. Furthermore, NO production (Figure 4) was significantly elevated in both HA and HA + EX groups after 24 h of incubation compared to control cultures. Overall, the suppressive effect of AMPNE was consistently observed across all tested parameters (Figure 1, Figure 2, Figure 3, Figure 4 and Figure 5).

Monocyte oxidative stress parameters, evaluated via NO accumulation and superoxide generation (Figure 6 and Figure 7), were highly significantly upregulated (*p* < 0.01) following exposure to HA relative to the unstimulated control (K). Specifically, HA exposure elicited a marked increase in monocyte NO release at both 24 h and 48 h time points, whereas baseline levels in control cultures reached 4.3 µM at 24 h and 6.7 µM at 48 h. Assessment of superoxide production similarly demonstrated a strong induction upon HA stimulation, peaking at 7.4 µM at 24 h compared to 4.2 µM in untreated controls. The inclusion of purified AMPNE as a functionalizing agent consistently attenuated the monocyte response. Specifically, HA + EX co-treatment highly significantly suppressed NO production at both 24 h (3.6 µM) and 48 h (4.2 µM) in comparison to unmodified HA. A parallel inhibitory effect was observed for superoxide generation, where HA + EX highly significantly attenuated reactive oxygen species levels at 24 h and 48 h, effectively restoring superoxide production to baseline control values. Overall, the suppressive effect of AMPNE on monocyte activation was consistently observed across both oxidative markers (Figure 6 and Figure 7).

## 4. Discussion

The early inflammatory response to implanted biomaterials critically determines the trajectory of subsequent tissue integration, remodeling, and long-term functional outcomes. Among innate immune cells, neutrophils constitute the first and most rapidly recruited leukocyte population at sites of implantation. Their activation is characterized by degranulation, oxidative burst, cytokine release, and extracellular trap formation, all of which shape the local immune microenvironment and influence downstream recruitment and polarization of monocytes/macrophages [23,24,25]. In fact, the initial interaction between the biomaterial and neutrophils is not an isolated event but rather the critical first step in a cascade that ultimately determines the phenotype and function of infiltrating monocytes, which are the key orchestrators of the subsequent chronic inflammatory and regenerative phases [26]. In this context, the present study provides mechanistic insight into the response of porcine peripheral blood neutrophils to hydroxyapatite and to HA functionalized with a purified peptide extract, focusing on enzymatic and oxidative markers of activation.

Exposure to unmodified HA induced a significant upregulation of elastase and MPO release, accompanied by enhanced production of reactive oxygen species and nitric oxide. These findings are consistent with previous evidence demonstrating that calcium phosphate–based ceramics, including HA, interact dynamically with circulating immune cells through rapid adsorption of plasma proteins, formation of a provisional matrix, and engagement of integrin-dependent signaling pathways [13,27]. The physicochemical characteristics of HA surfaces such as crystallinity, porosity, surface roughness, and ionic dissolution have been shown to modulate immune cell adhesion and activation, potentially triggering oxidative burst through NADPH oxidase activation [28,29].

The observed increase in MPO release reflects degranulation of azurophilic granules and is widely considered a hallmark of acute neutrophil activation [23]. MPO-derived oxidants, including hypochlorous acid, contribute to antimicrobial defense but may also induce collateral oxidative damage to surrounding tissues and extracellular matrix components when excessively produced [24]. Similarly, neutrophil elastase, a serine protease stored in primary granules, plays a role in matrix remodeling and antimicrobial activity; however, uncontrolled elastase release has been implicated in tissue degradation and impaired wound healing [23,25]. Therefore, the elevated enzymatic activity detected in HA-exposed neutrophils suggests a pronounced pro-inflammatory phenotype at the material interface.

The enhancement of ROS and NO generation further supports the notion of oxidative activation in response to unmodified HA. ROS production is an essential component of innate immunity, but persistent oxidative stress at implant sites is associated with lipid peroxidation, protein modification, and DNA damage, potentially contributing to chronic inflammation and fibrotic encapsulation [29]. Nitric oxide, synthesized by inducible nitric oxide synthase (iNOS) in activated neutrophils, exerts antimicrobial and signalling functions; however, excessive NO may react with superoxide to form peroxynitrite, amplifying oxidative tissue injury [24]. Thus, the combined elevation of ROS and NO observed in the HA group indicates an amplified redox imbalance that could negatively influence early osseointegration if not adequately resolved.

In contrast, functionalization of HA with the purified peptide extract (HA + EX) resulted in attenuation of elastase and MPO release and a reduction in ROS and NO generation, particularly at the 24 h time point. This suggests that peptide modification modulates neutrophil activation at the cell–material interface. Surface-bound bioactive peptides are increasingly recognized as immuno-instructive cues capable of altering protein adsorption profiles and modulating integrin engagement, toll-like receptor (TLR) signaling, and downstream transcriptional programs [24,30]. By modifying the interfacial microenvironment, peptides may dampen excessive oxidative burst while preserving essential defensive functions.

The temporal aspect of the response is particularly noteworthy. While early neutrophil activation is necessary for debris clearance and microbial protection, prolonged activation may impair regenerative processes [23,25]. The more pronounced difference between HA and HA + EX at 24 h suggests that peptide functionalization may not completely prevent initial activation but rather facilitates a more rapid transition toward resolution. This is consistent with contemporary concepts of neutrophil plasticity and heterogeneity. Neutrophils are no longer regarded as terminally differentiated, short-lived effector cells; instead, they exhibit functional diversity and can adopt phenotypes ranging from highly pro-inflammatory (N1-like) to pro-resolving or regenerative (N2-like) states depending on microenvironmental signals [25]. Surface modifications that influence early neutrophil signaling may therefore have downstream consequences for macrophage polarization and the overall osteoimmune response.

By tempering the early oxidative and enzymatic burst, the HA + EX surface may not only promote a more pro-resolving neutrophil phenotype but also create a microenvironment that is conducive to the recruitment of monocytes and their subsequent polarization toward a pro-regenerative (M2-like) state. Monocytes are highly responsive to the local cytokine milieu; signals such as interleukin-4 (IL-4), interleukin-10 (IL-10), and low levels of ROS, which are more likely to prevail in the HA + EX condition, can guide their differentiation into M2 macrophages. These cells are characterized by the production of anti-inflammatory cytokines (e.g., transforming growth factor-beta (TGF-β), IL-10), the secretion of pro-angiogenic and pro-fibrotic factors, and the support of extracellular matrix deposition and tissue remodeling [31]. Therefore, the attenuated neutrophil activation observed in the HA + EX group represents a potential mechanism for steering monocyte/macrophage polarization toward a regenerative phenotype, a finding that is central to the field of immunomodulatory biomaterials.

From an osteoimmunological perspective, the interplay between early immune responders and bone-forming processes is increasingly appreciated. Neutrophil-derived cytokines, proteases, and reactive oxygen species can modulate signaling pathways relevant to bone remodeling, where excessive oxidative stress is known to impair osteogenic differentiation and extracellular matrix mineralization in vitro [18,30]. In this context, the attenuated ROS release and enzymatic activity observed in the HA + EX group suggest a reduced acute pro-inflammatory load, which may provide a more favorable cellular microenvironment for subsequent osteoblastic responses. Furthermore, monocyte-derived macrophages play a key role in orchestrating tissue repair, where pro-inflammatory (M1-like) signaling is often linked to sustained tissue degradation, whereas pro-healing (M2-like) phenotypes secrete osteoinductive factors (e.g., BMP-2, VEGF) involved in tissue regeneration [32,33]. Although direct macrophage polarization marker panels and in vivo osseointegration were not evaluated in the present study, our in vitro observations of attenuated leukocyte activation indicate that the HA + EX surface has the potential to minimize excessive early inflammation. Whether this early immunomodulatory effect translates into enhanced M2 polarization or improved osseointegration remains an attractive hypothesis that warrants confirmation in future in vivo models.

Another important consideration is the role of biomaterial-driven immune modulation in preventing chronic foreign body reactions. Persistent neutrophil activation can contribute to sustained recruitment of monocytes and the formation of foreign body giant cells, ultimately leading to fibrotic encapsulation [29]. By attenuating early neutrophil activation, peptide-functionalized HA may indirectly reduce the risk of maladaptive chronic inflammation. This aligns with the emerging paradigm of immunomodulatory biomaterials, which emphasizes the deliberate design of surfaces capable of directing host immune responses toward constructive remodelling rather than pathological inflammation [24,30].

It should also be emphasized that the present study utilized porcine neutrophils, which represent a translationally relevant large-animal model due to physiological similarities to the human immune system. Nevertheless, in vitro monoculture systems inherently lack the complexity of in vivo inflammatory networks. Cross-talk between neutrophils and macrophages, endothelial cells, mesenchymal stem cells, and osteoprogenitors critically shapes the regenerative milieu. Furthermore, processes such as neutrophil extracellular trap formation, apoptosis, and efferocytosis were not evaluated in the present investigation and may further modulate biomaterial integration [23,25]. Future research should incorporate co-culture systems, advanced 3D models, and in vivo validation to determine whether the immunomodulatory effects observed here translate into improved osseointegration and mechanical stability.

To evaluate the practical rationale for incorporating a chromatography-based downstream purification step, the present results must be contextualized alongside our previous baseline investigation on crude porcine neutrophil peptide extracts [13]. In our earlier work, the unpurified extract exhibited broad immunomodulatory effects; however, its activity was partially confounded by background cellular debris and non-specific protein contaminants present in the crude matrix. By applying size-exclusion gel chromatography in the current protocol, we successfully eliminated these interfering high-molecular-weight components, enriching the active cathelicidin and protegrin fraction. Comparing the functional outcomes reveals a clear biological advantage of the purified preparation: while the crude extract in our previous study conferred only a partial attenuation of biomaterial-induced oxidative stress, the purified AMPNE in the current study achieved a significantly stronger, highly consistent downregulation of both primary granule exocytosis (elastase, MPO) and ROS/RNS production in neutrophils and monocytes. This superior inhibitory efficacy confirms that isolating the bioactive peptide core eliminates suppressive or pro-inflammatory matrix noise, thereby enhancing targeted bioactivity, functional specificity, and overall anti-inflammatory potency at the biomaterial interface.

A critical aspect of implementing the newly developed purification method was verifying whether the isolated AMPNE particles successfully preserved their functional properties. Post-purification analysis revealed an enhancement in the observed effect due to the removal of matrix impurities and modulation of the biological response. This confirms that the purification process does not compromise the structural and functional integrity of AMPNE components. Furthermore, the observed strengthening of the effect demonstrates that the biological activity is intrinsically linked to the targeted particles themselves rather than nonspecific constituents of the crude extract, thereby successfully validating the efficacy of the proposed purification approach.

Overall, the data indicate that peptide functionalization of hydroxyapatite modifies early neutrophil behavior by attenuating excessive enzymatic and oxidative activation. This immunomodulatory effect is likely to have a profound influence on the subsequent population of infiltrating monocytes, potentially steering their differentiation towards a pro-regenerative phenotype and thereby creating a more favorable environment for tissue healing and implant integration. These findings contribute to the expanding field of immunoengineering, where modulation of innate immune responses is recognized as a cornerstone for optimizing biomaterial performance.

## 5. Conclusions

The present study provides detailed evidence that hydroxyapatite induces a robust activation of porcine neutrophils characterized by increased degranulation and enhanced production of reactive oxygen and nitrogen species, reflecting a pronounced early inflammatory response at the biomaterial interface. Such activation is consistent with established models of neutrophil-driven acute inflammation and underscores the importance of controlling early innate immune events following implantation. This strong early response is a critical determinant of the subsequent inflammatory cascade, as it directly influences the recruitment and functional polarization of monocytes, which are key drivers of chronic inflammation and tissue repair. Functionalization of hydroxyapatite with a purified peptide extract significantly attenuated these activation markers, indicating that surface modification can effectively modulate neutrophil behavior without completely abolishing physiological immune responsiveness.

The reduction in elastase and MPO release, together with decreased ROS and NO generation, suggests that peptide-functionalized HA promotes a more balanced redox and enzymatic profile that may limit collateral tissue damage and support a microenvironment conducive to tissue regeneration. Since early neutrophil signaling is crucial for attracting and activating macrophages and bone-forming processes, this adjustment could significantly affect how well biomaterials integrate over time and their overall effectiveness. The ability of the HA + EX surface to curb the initial neutrophil burst, potentially steering the monocyte/macrophage response toward a healing phenotype, represents a promising strategy for promoting constructive tissue remodeling and preventing chronic fibrosis. These findings support the concept that rationally designed peptide-based surface modifications represent a promising strategy for developing next-generation immunomodulatory biomaterials aimed at optimizing host–material interactions and enhancing regenerative success. Further in vivo studies are warranted to confirm whether the observed in vitro immunomodulatory effects translate into improved osseointegration and reduced inflammatory complications in clinically relevant models.

## Figures and Tables

**Figure 1:**
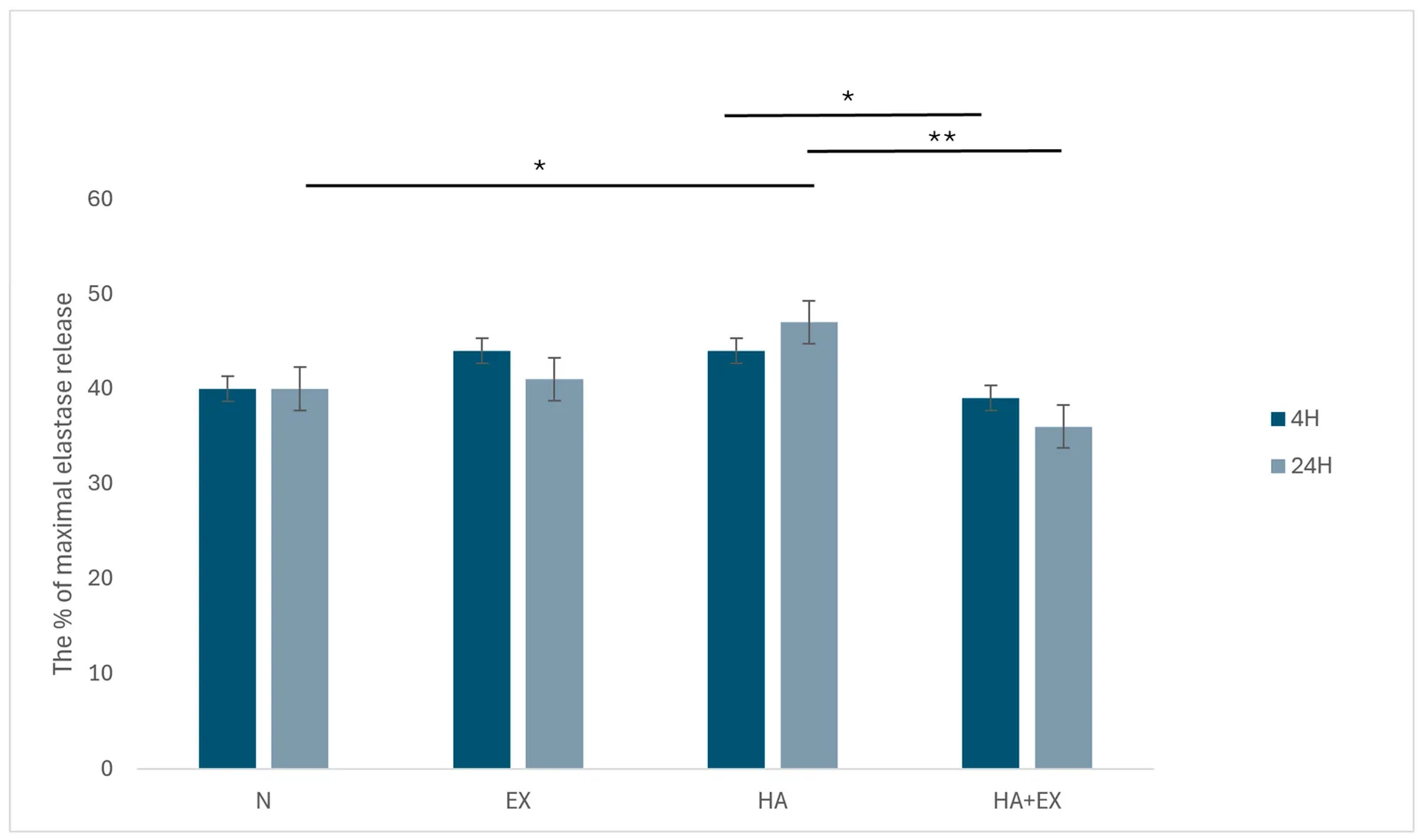
Relative elastase secretion expressed as a percentage of maximum response. Experimental conditions: N—untreated porcine neutrophils; EX—neutrophils stimulated with purified porcine AMPNE; HA—neutrophils exposed to hydroxyapatite; HA + EX—neutrophils co-treated with hydroxyapatite and purified porcine AMPNE. Values represent the mean ± standard deviation from three independent replicates. Statistical significance was evaluated using a one-tailed paired Student’s *t*-test (* *p* < 0.05, ** *p* < 0.01). Horizontal lines indicate statistically significant comparisons between the connected groups.

**Figure 2:**
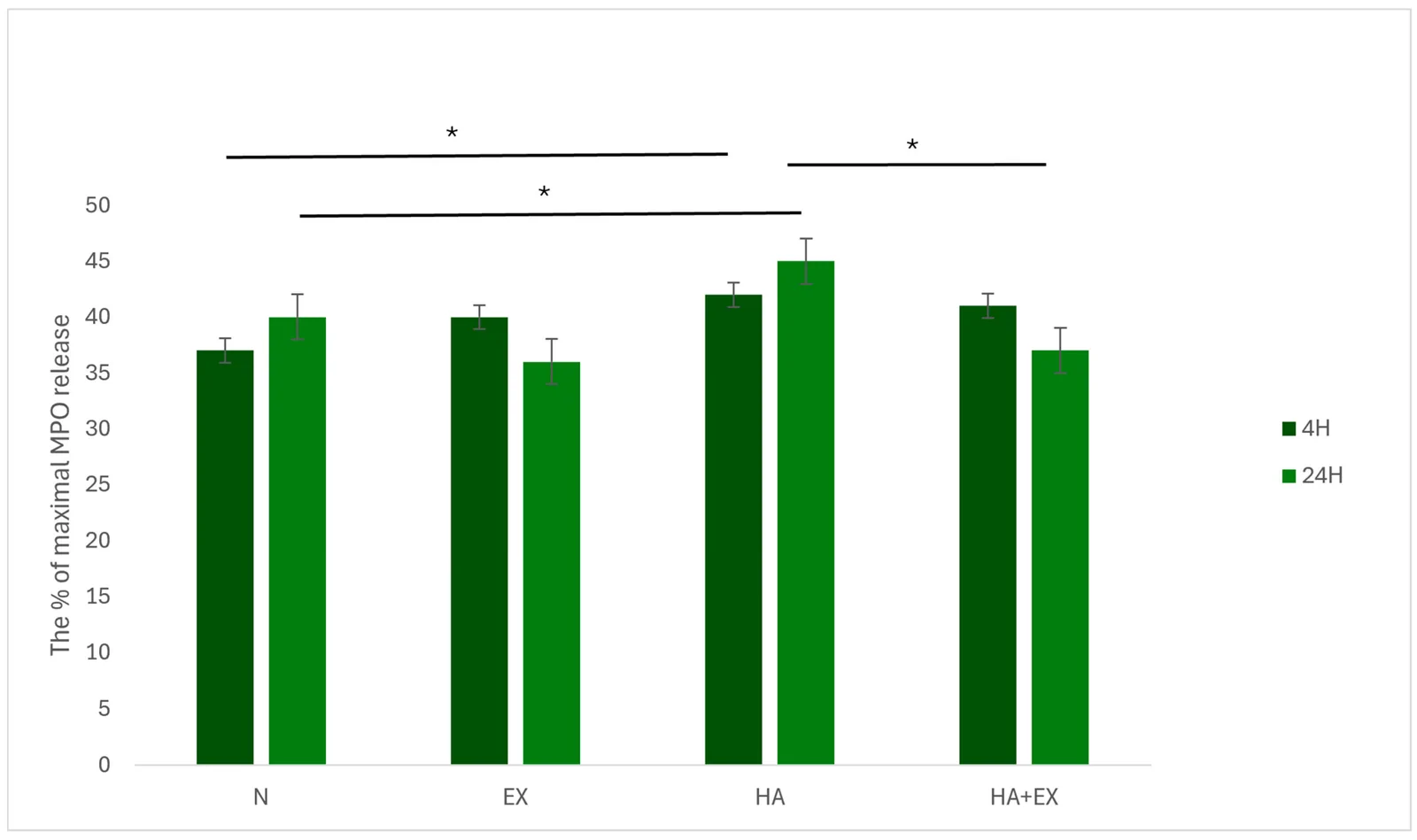
Relative MPO secretion expressed as a percentage of maximum response. Experimental conditions: N—untreated porcine neutrophils; EX—neutrophils stimulated with purified porcine AMPNE; HA—neutrophils exposed to hydroxyapatite; HA + EX—neutrophils co-treated with hydroxyapatite and purified porcine AMPNE. Values represent the mean ± standard deviation from three independent replicates. Statistical significance was evaluated using a one-tailed paired Student’s *t*-test (* *p* < 0.05). Horizontal lines indicate statistically significant comparisons between the connected groups.

**Figure 3:**
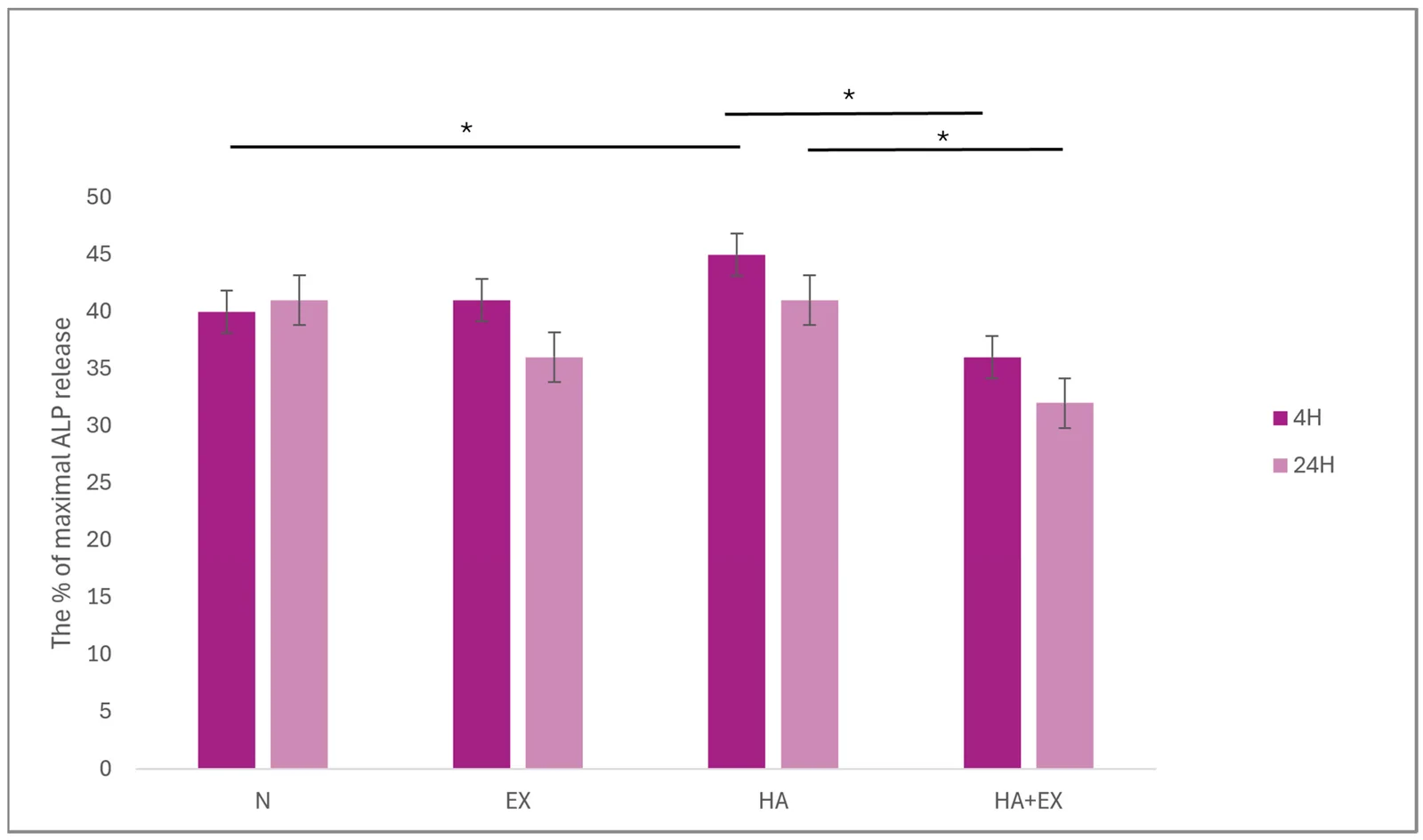
Relative ALP secretion expressed as a percentage of maximum response. Experimental conditions: N—untreated porcine neutrophils; EX—neutrophils stimulated with purified porcine AMPNE; HA—neutrophils exposed to hydroxyapatite; HA + EX—neutrophils co-treated with hydroxyapatite and purified porcine AMPNE. Values represent the mean ± standard deviation from three independent replicates. Statistical significance was evaluated using a one-tailed paired Student’s *t*-test (* *p* < 0.05). Horizontal lines indicate statistically significant comparisons between the groups spanned by each line.

**Figure 4:**
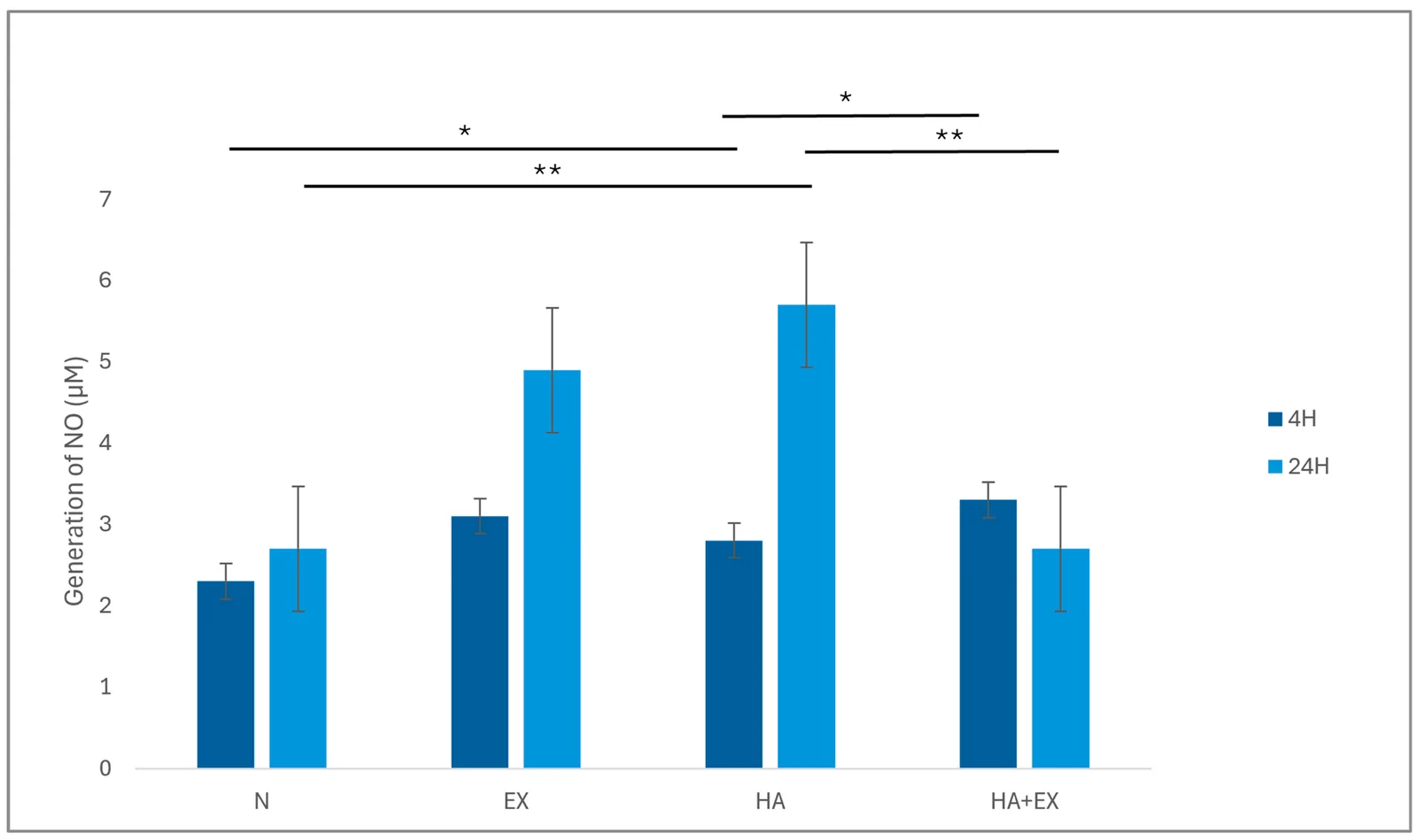
NO accumulation (µM) in culture supernatants. Experimental conditions: N—untreated porcine neutrophils; EX—neutrophils stimulated with purified porcine AMPNE; HA—neutrophils exposed to hydroxyapatite; HA + EX—neutrophils co-treated with hydroxyapatite and purified porcine AMPNE. Values represent the mean ± standard deviation from three independent replicates. Statistical significance was evaluated using a one-tailed paired Student’s *t*-test (* *p* < 0.05, ** *p* < 0.01). Horizontal lines indicate statistically significant comparisons between the groups spanned by each line.

**Figure 5:**
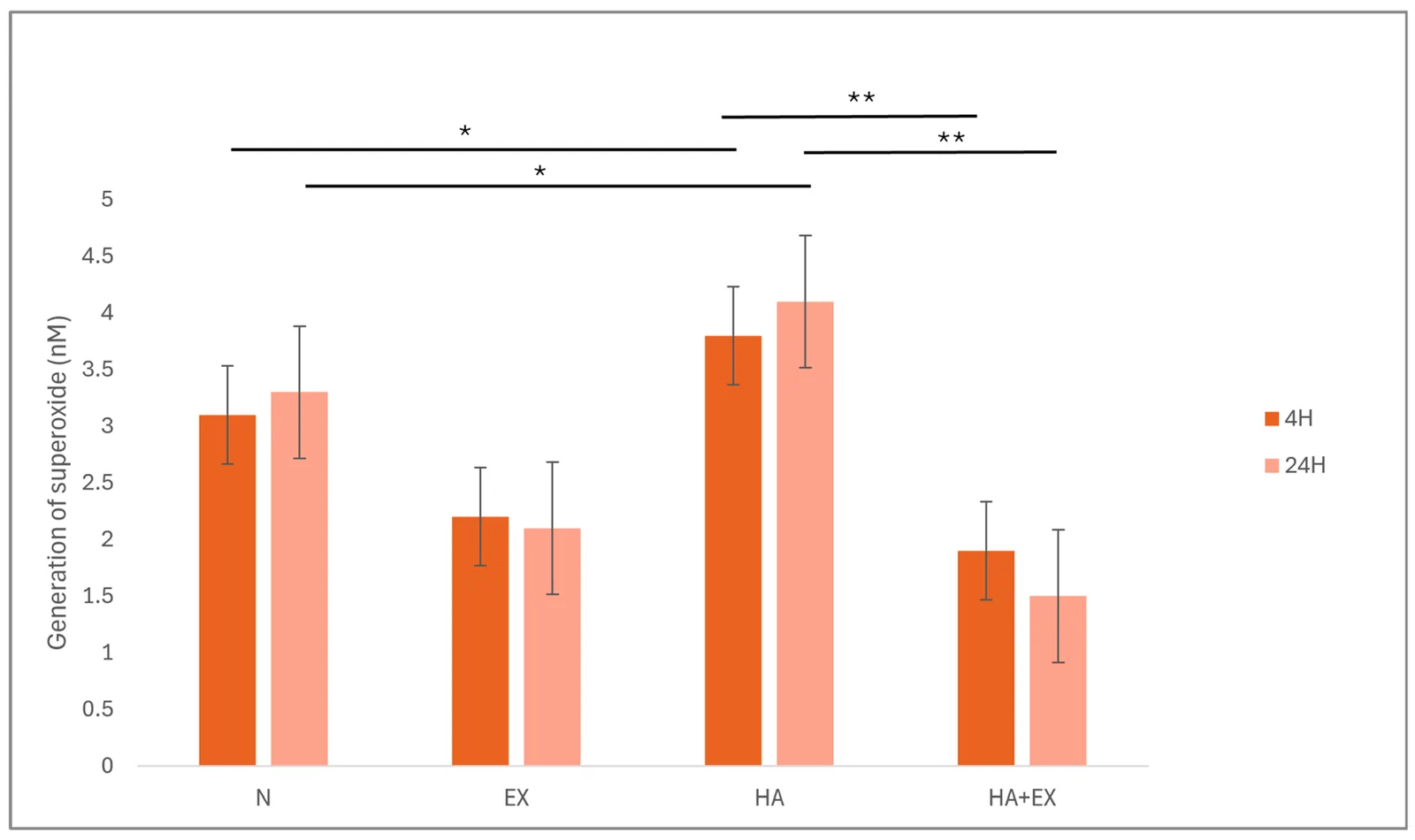
Generation of superoxide (nM) in culture supernatants. Experimental conditions: N—untreated porcine neutrophils; EX—neutrophils stimulated with purified porcine AMPNE; HA—neutrophils exposed to hydroxyapatite; HA + EX—neutrophils co-treated with hydroxyapatite and purified porcine AMPNE. Values represent the mean ± standard deviation from three independent replicates. Statistical significance was evaluated using a one-tailed paired Student’s *t*-test (* *p* < 0.05, ** *p* < 0.01). Horizontal lines indicate statistically significant comparisons between the groups spanned by each line.

**Figure 6:**
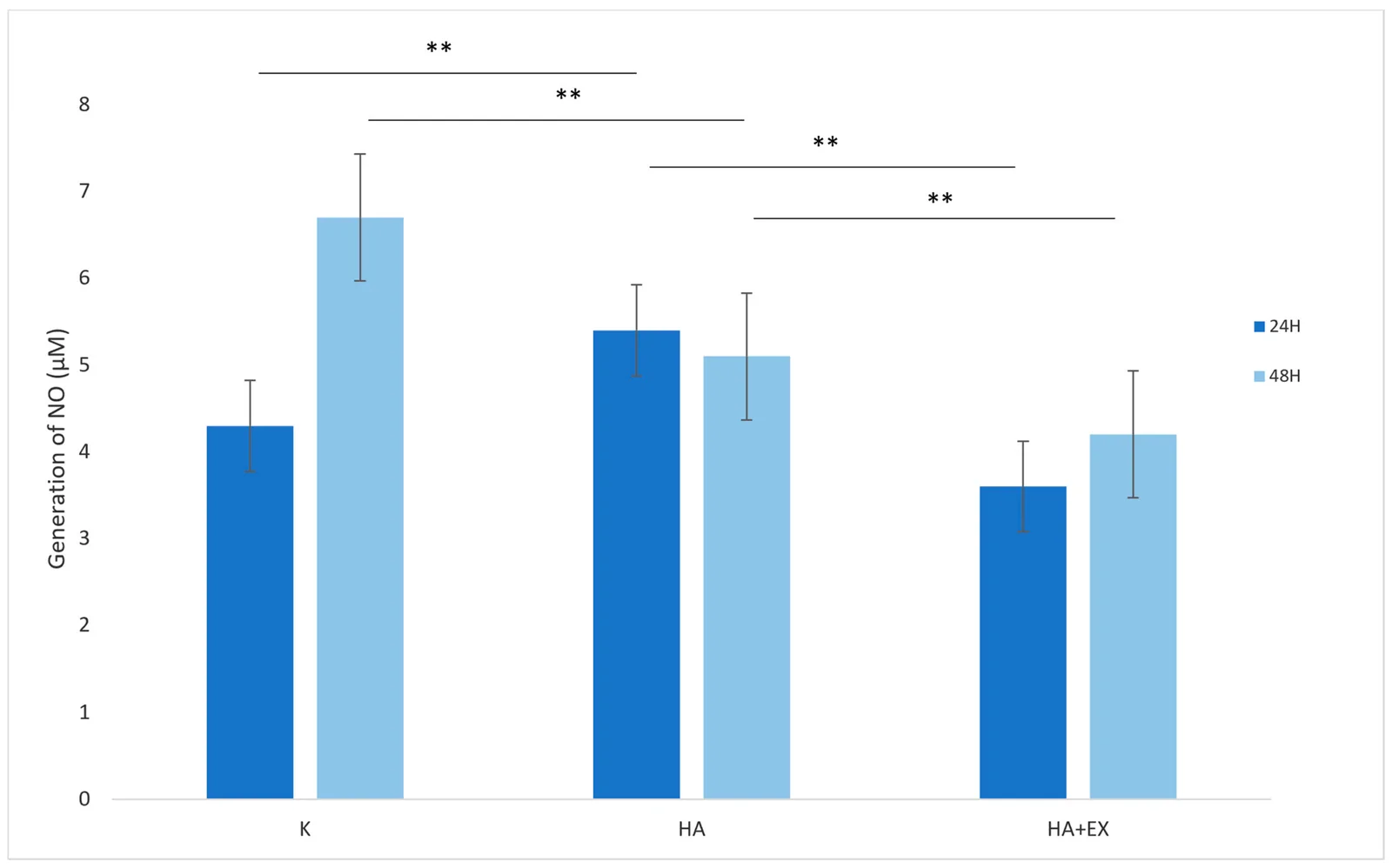
NO accumulation (µM) in culture supernatants. Experimental conditions: K—untreated porcine monocytes; HA—monocytes exposed to hydroxyapatite; HA + EX—monocytes co-treated with hydroxyapatite and purified porcine AMPNE. Values represent the mean ± standard deviation from three independent replicates. Statistical significance was evaluated using a one-tailed paired Student’s *t*-test (** *p* < 0.01). Horizontal lines indicate statistically significant comparisons between the groups spanned by each line.

**Figure 7:**
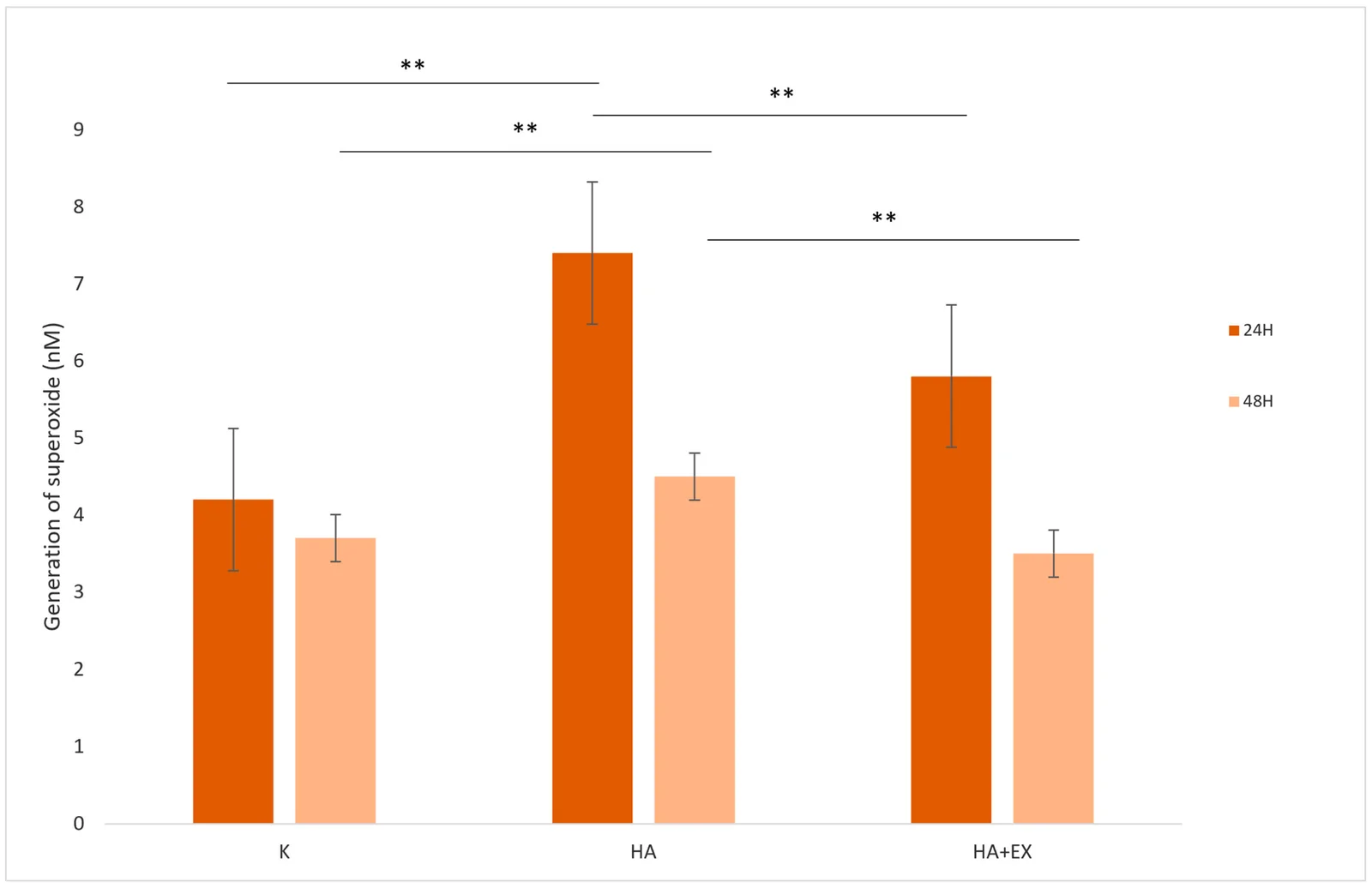
Generation of superoxide (nM) in culture supernatants. Experimental conditions: K—untreated porcine monocytes; HA—monocytes exposed to hydroxyapatite; HA + EX—monocytes co-treated with hydroxyapatite and purified porcine AMPNE. Values represent the mean ± standard deviation from three independent replicates. Statistical significance was evaluated using a one-tailed paired Student’s *t*-test (** *p* < 0.01). Horizontal lines indicate statistically significant comparisons between the groups spanned by each line.

## Data Availability

The original contributions presented in this study are included in the article. Further inquiries can be directed to the corresponding author.
